# The human papillomavirus confers radiosensitivity in oropharyngeal cancer cells by enhancing DNA double strand break

**DOI:** 10.18632/oncotarget.27535

**Published:** 2020-04-21

**Authors:** Mei Zhang, Angela M. Hong

**Affiliations:** ^1^Faculty of Medicine and Health, Central Clinical School, The University of Sydney, NSW, Australia; ^2^Department of Radiation Oncology, Chris O’Brien Lifehouse, Camperdown, NSW, Australia

**Keywords:** radiosensitivity, oropharyngeal cancer, human papillomavirus, double-strand break, radiobiology

## Abstract

**Background:** Patients with Human papillomavirus (HPV)-positive oropharyngeal squamous cell carcinoma (OPSCC) has better outcomes than those with HPV-negative OPSCC. This may be related to its enhanced radiosensitivity. This study examined the effect of HPV and its E6 oncoprotein on the morphology, radiosensitivity, and repair of radiation-induced DNA damage.

**Materials and Methods:** HPV-negative UM-SCC4 with and without transfection of HPV E6 oncoprotein, HPV-negative UPCI-SCC-089 and HPV-positive UPCI-SCC-099 cell lines were used in this study. The radiosensitivity and morphological changes after radiation were determined by clonogenic assay. Radiation-induced double-strand breaks in the DNA was measured by γ-H2AX foci immunofluorescent assay.

**Results:** The survival fraction after 10 Gy was significantly lower for the HPV-positive SCC-099 cells than for the HPV-negative cells (*p* = 0.03). The levels of γ-H2AX foci formation and retention were time and cell line-dependent. The γ-H2AX level started to increase at 1 hour and peaked at 4 hours after 10 Gy radiation in the HPV-negative SCC-089 and UM-SCC4 cells before reducing to negligible level (*p* = 0.0001). In contrast, the HPV-positive UPCI-SCC-099 cells displayed persistent γ-H2AX activity; the expression of γ-H2AX remained high at 48 hours post radiation (*p* = 0.001). Transfection with the E6 oncoprotein prolonged γ-H2AX formation up to 24 hours in HPV-negative SCC4 cells. HPV-positive SCC-099 cells were more likely to show the classical apoptotic changes of increased cell thickness and increased motility after radiation.

**Conclusions:** This *in vitro* study confirmed that HPV-positive OPSCC was more radiosensitive. Transfection with the E6 oncoprotein enhanced the radiosensitivity in HPV-negative OPSCC by impairing the DNA repair mechanism and enhancing apoptotic cell death.

## INTRODUCTION

Human papillomavirus (HPV)-positive oropharyngeal squamous cell carcinoma (OPSCC) is clinically and biologically distinct from smoking-related (HPV-negative) OPSCC. The newly published TNM classification (eighth edition) includes HPV status in the staging of OPSCC [1]. Patients with HPV-positive OPSCC tend to be younger with more advanced nodal disease at diagnosis but have better outcomes [2–6]. The overall better prognosis seen in HPV-positive OPSCC may be related to the disease’s response to radiation therapy.

Radiation therapy plays an important role in the management of OPSCC, either as definitive therapy or as adjuvant therapy after surgery. The main mechanism of radiation therapy is direct DNA damage, including single-strand break and double-strand break (DSB). The biological response to DSB induction is largely determined by DSB repair processes. In response to DSB induction, histone H2AX located around the break site is rapidly phosphorylated (γ-H2AX) on serine 139 by members of the PI3 kinase family. Most studies have suggested that persistence of γ-H2AX levels after radiation is a sign of lethal damage by radiation [7–10]. The ability to repair this DNA damage varies between different cancer types and normal tissue and correlates with variation in radiosensitivity. Recent *in vitro* studies have suggested that HPV-positive OPSCC may impair DNA repair mechanisms [11–14].

Cellular response to radiation treatment can be observed with a label-free dynamic HoloMonitor, which allows non-invasive visualization and live cell analysis of radiation responses [15] and the migration potential of cancer cells [16]. This study used HoloMonitor to examine the effect of HPV and its E6 oncoprotein on the morphology, radiosensitivity, and repair of radiation-induced DNA DSB of OPSCC cell lines.

## RESULTS

### HPV-positive OPSCC cells are more radiosensitive than HPV-negative OPSCC by proliferation test and clonogenic survival

**Figure 1:**
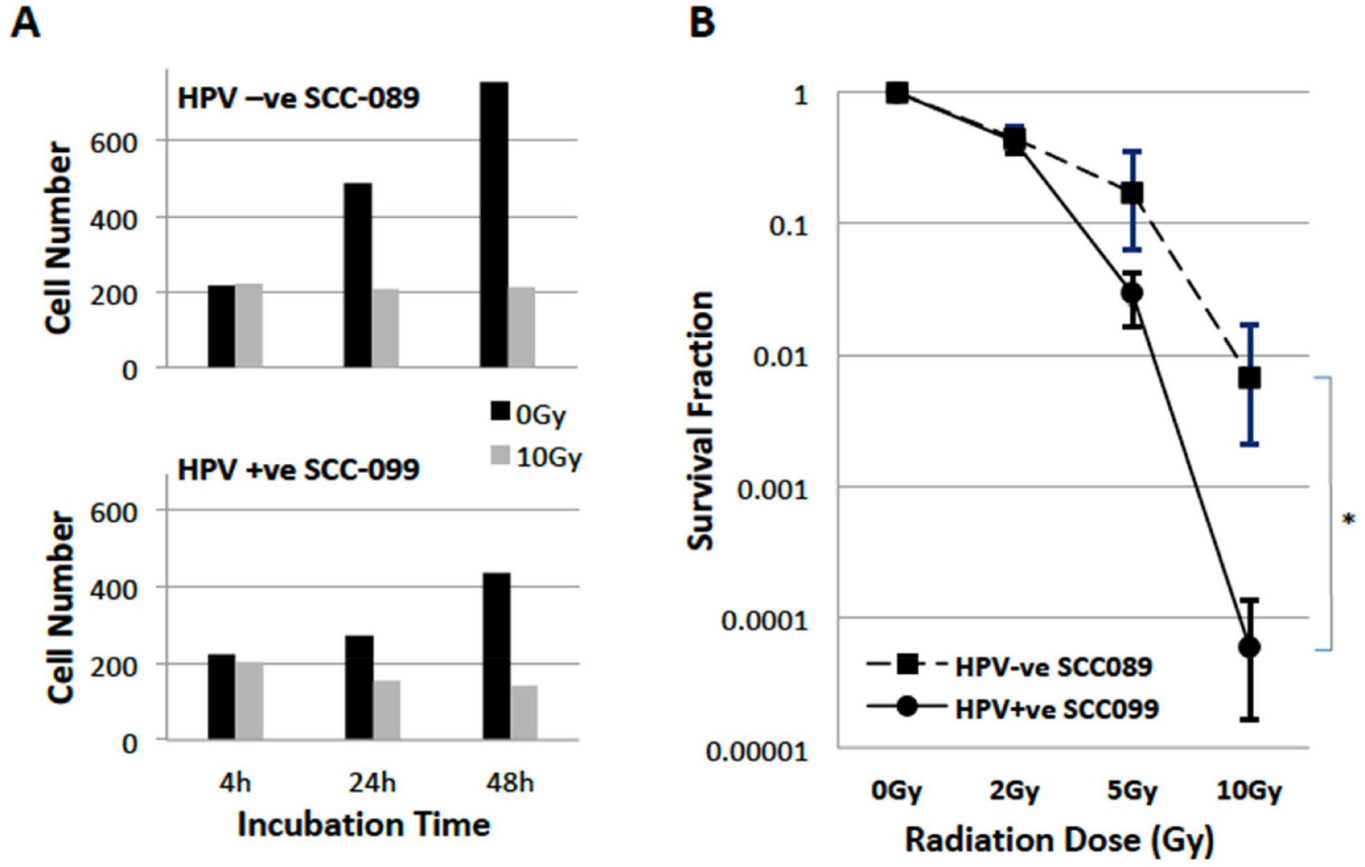
Radiosensitivity of HPV-SCC-089 and HPV+SCC-099 cells. (**A**) Cell proliferation inhibition recorded by HoloMonitor for 48 hrs, cell number counting comparing unirradiated control (black column) and 10 Gy irradiated (grey column). (**B**) Clonogenic survival curves of HPV-SCC-089 and HPV+SCC-099 cells. ^*^ *P* = 0.03.

Figure 1A showed the cell number changes recorded by live cell HoloMonitor for 48 hours. Cell doubling time was about 24 hours and 48 hours for unirradiated UPCI-SCC-089 and UPCI-SCC-099 respectively. After 10 Gy of irradiation, UPCI-SCC-099 showed a 20% reduction in proliferation. However, UPCI-SCC-089 did not show a change in cell number after 10 Gy radiation. Figure 1B showed the radiation survival curve of these two cell lines after single doses of 2 to 10 Gy radiation. The survival after 2 Gy (SF2) was 0.45 and 0.43 (p = ns); survival after 10 Gy was 0.0067 and 0.000057 (*P* = 0.03) for UPCI-SCC-089 and UPCI-SCC-099 respectively.

### Distinct radiation induced morphological changes

**Figure 2:**
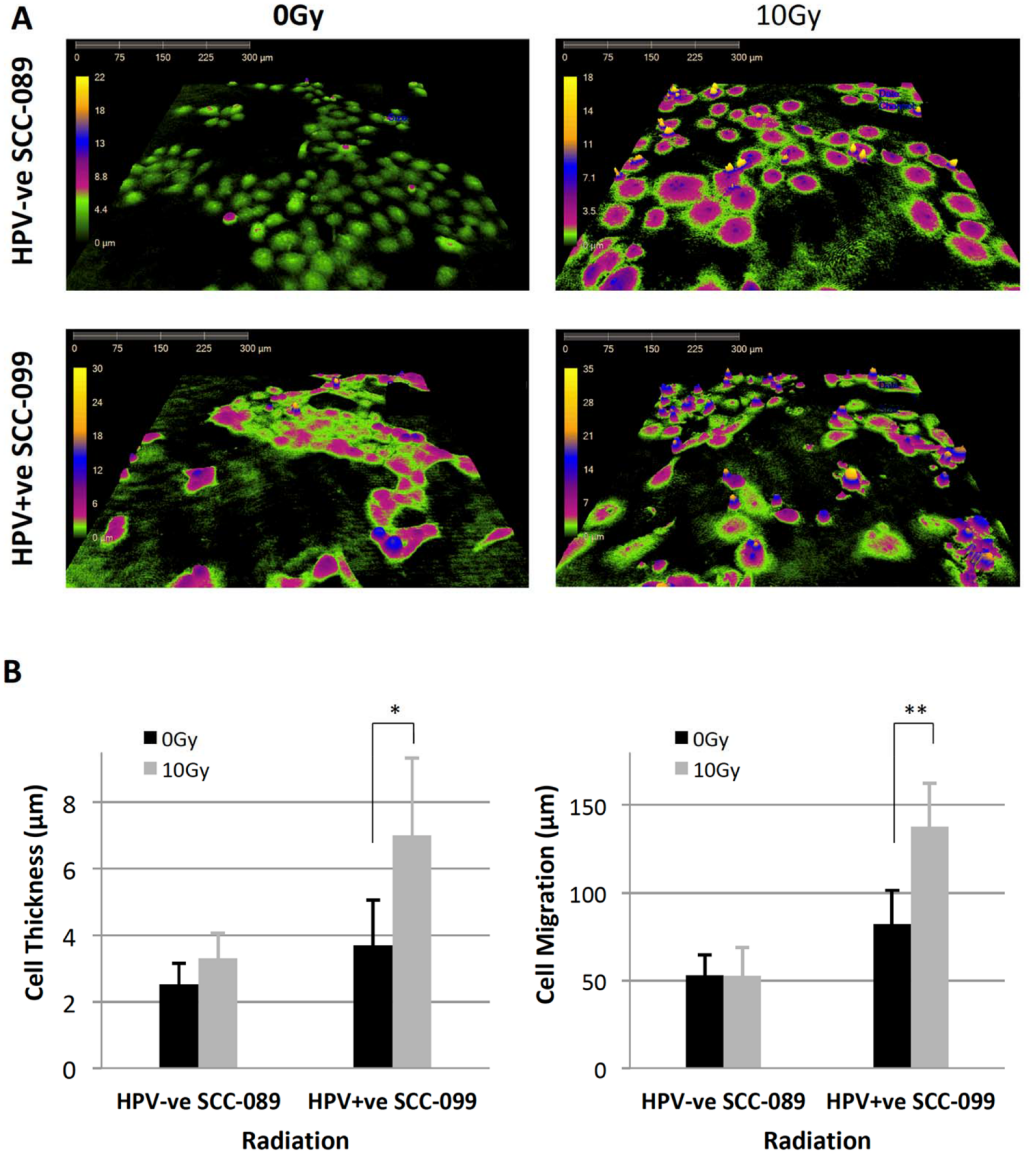
Radiation induced morphological changes on HPV-SCC-089 and HPV+SCC-099 by Holographic microscopy. (**A**) Representative view of cell size, thickness and confluence at 30 hours after plating with or without irradiation. (**B**) Left: Column graph demonstrates the quantitative analyze of cell volume and cell thickness. ^*^ *P* = 0.0001, ^**^ *P* = 0.005. Right: Quantitative analyze of the cell migration. ^*^ *P* = 0.0003.

Radiation caused distinct morphological changes in HPV-positive UPCI-SCC-099 cells in comparison to HPV-negative UPCI-SCC-089 as detected by the Digital live cell HoloMonitor M4 (Supplementary Videos 1 and 2). At 30 hours post irradiation (Figure 2A), the UPCI-SCC-089 cells (top row) exhibited cell flattening and enlargement whilst the UPCI-SCC-099 cells demonstrated an increase in cell thickness. The quantitative analysis of all tracked cells confirmed this observation. As shown in Figure 2B at 48 hours after irradiation, UPCI-SCC-099 showed a significant increase in the thickness of the individual cell and cell migration.

**Figure 3:**
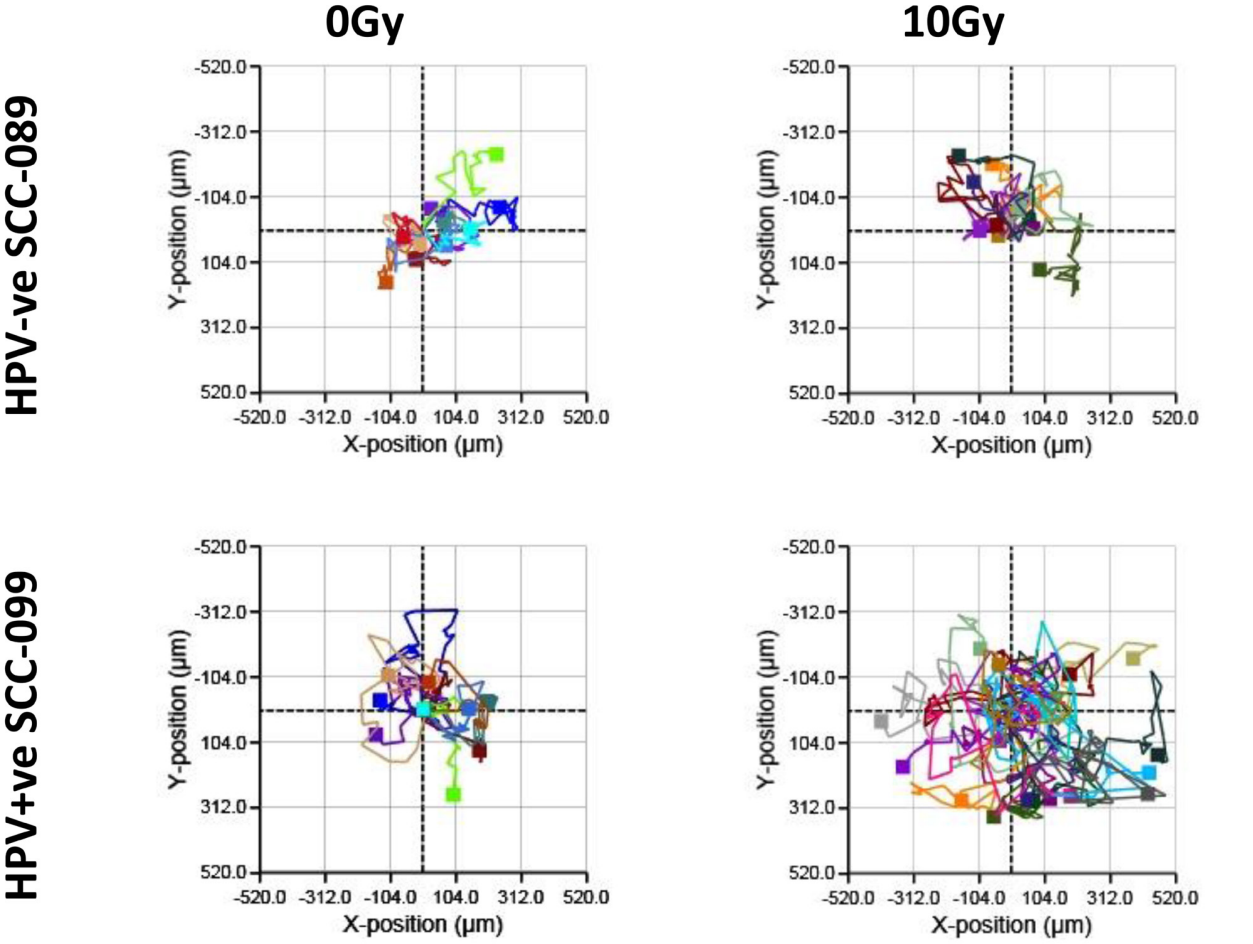
Cell movement in HPV-SCC-089 and HPV+SCC-099 by Holographic microscopy. Cell movement plot comparing untreated and 10 Gy irradiated cells in 48 hours tracking period.

The motility of UPCI-SCC-089 and UPCI-SCC-099 cells were similar at baseline (Figure 3). At 48 hours after 10 Gy of radiation, there was enhanced motility of the UPCI-SCC-099 cells (right panel Figure 3 and Supplementary Video 2). The quantitative analysis (Figure 2B), demonstrated a significant increase in the average cell movement in HPV-positive UPCI-SCC-099 from 82 μm to 134 μm after irradiation (*p* = 0.0003). In contrast, radiation did not cause any significant change in the movement of HPV-negative UPCI-SCC089 (53 μm for control and irradiated cells).

### Time and cell line dependent DNA damage repair ability

**Figure 4:**
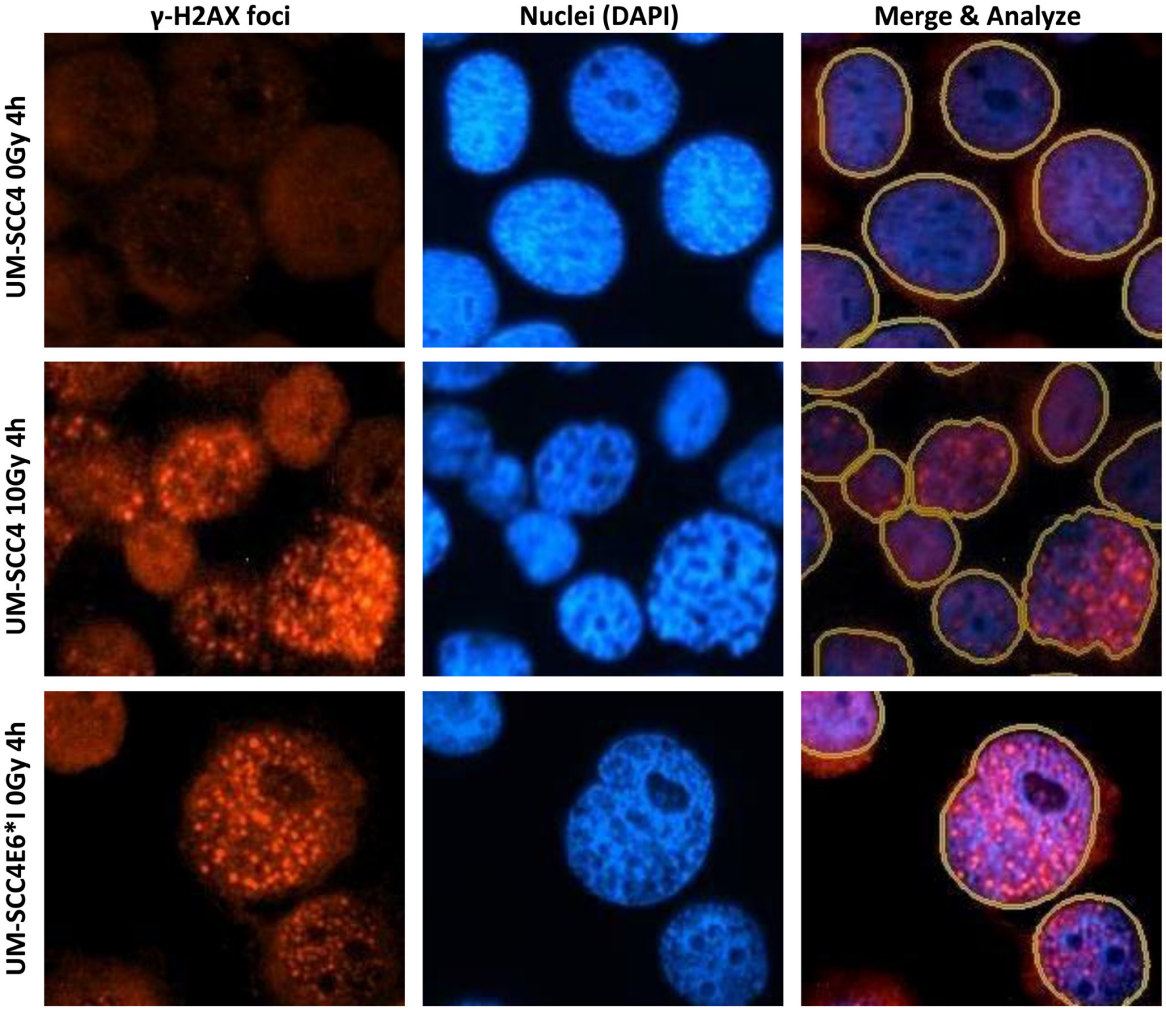
Examples of γ-H2AX foci staining of UM-SCC4 and UM-SCC4 E6*I cells. Left panel: γ-H2AX foci staining (Red signal); Middle panel: DAPI nucleus counter staining (Blue staining); Right panel: merged image of γ-H2AX foci and DAPI. The marking of each nucleus border was shown as yellow circle. In each sample, 200–800 cells from at least 5 random microscopic views were used for quantitative assessment with Gen5 software. Magnification: ×20.

**Figure 5:**
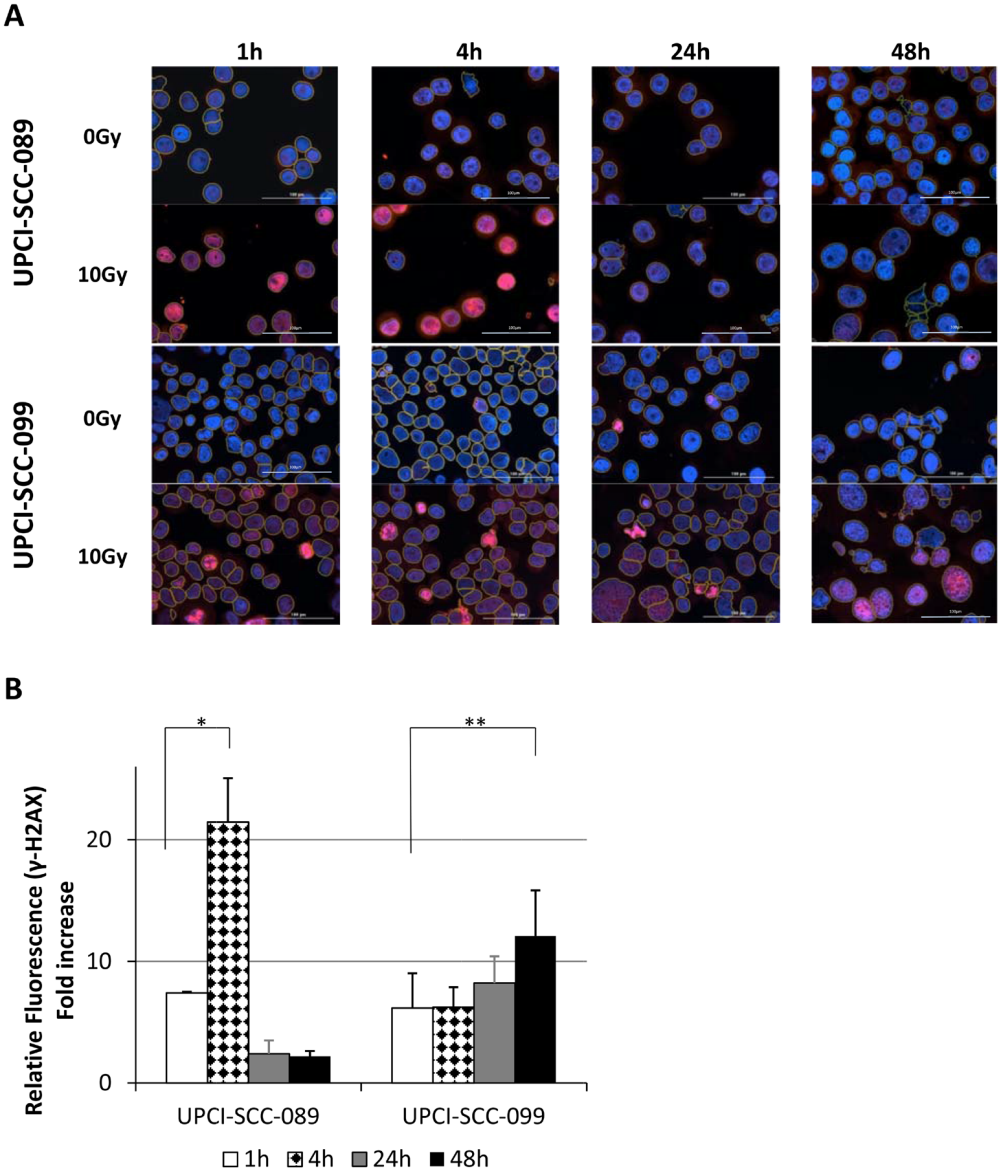
Radiation induced DNA damage measured by γ-H2AX foci formation at specified time point after 10 Gy. (**A**) Representative images of HPV-SCC-089 and HPV+SCC-099 with merged images of γ-H2AX, DAPI and nucleus marking. Magnification: ×20, scale bar = 100 μm. (**B**) Column graph showing quantitative analyze of γ-H2AX foci by relative fold change of fluorescent intensity in HPV-SCC-089 and HPV+SCC-099. ^*^ *P* = 0.0001, ^**^ *P* = 0.001.

By comparing UPCI-SCC-089 and UPCI-SCC-099, the γ-H2AX foci formation was not only time dependent but also cell line dependent. Examples of γ-H2AX foci staining and the marking of an individual cell nucleus from Gen5 software are shown in Figure 4. As shown in Figure 5A, strong red γ-H2AX signal was detected at 1 hour and 4 hours post 10 Gy radiation in both cell lines. The signal decreased dramatically in UPCI-SCC-089 after 4 hours. In contrast, the positive staining was retained up to 48 hours in UPCI-SCC-099 cells. Figure 5B showed the quantitative measurement of the intensity of γ-H2AX. The γ-H2AX foci in HPV-negative cell UPCI-SCC-089 cell line started to increase 1 hour after 10 Gy of radiation, peaked at 4 hours then significantly reduced to a negligible level (*P* = 0.0001). In contrast, γ-H2AX foci in HPV-positive UPCI-SCC-099 accumulated continuously from 1 hour post radiation and the intensity level remained high at 48 hours post radiation (*P* = 0.001).

**Figure 6:**
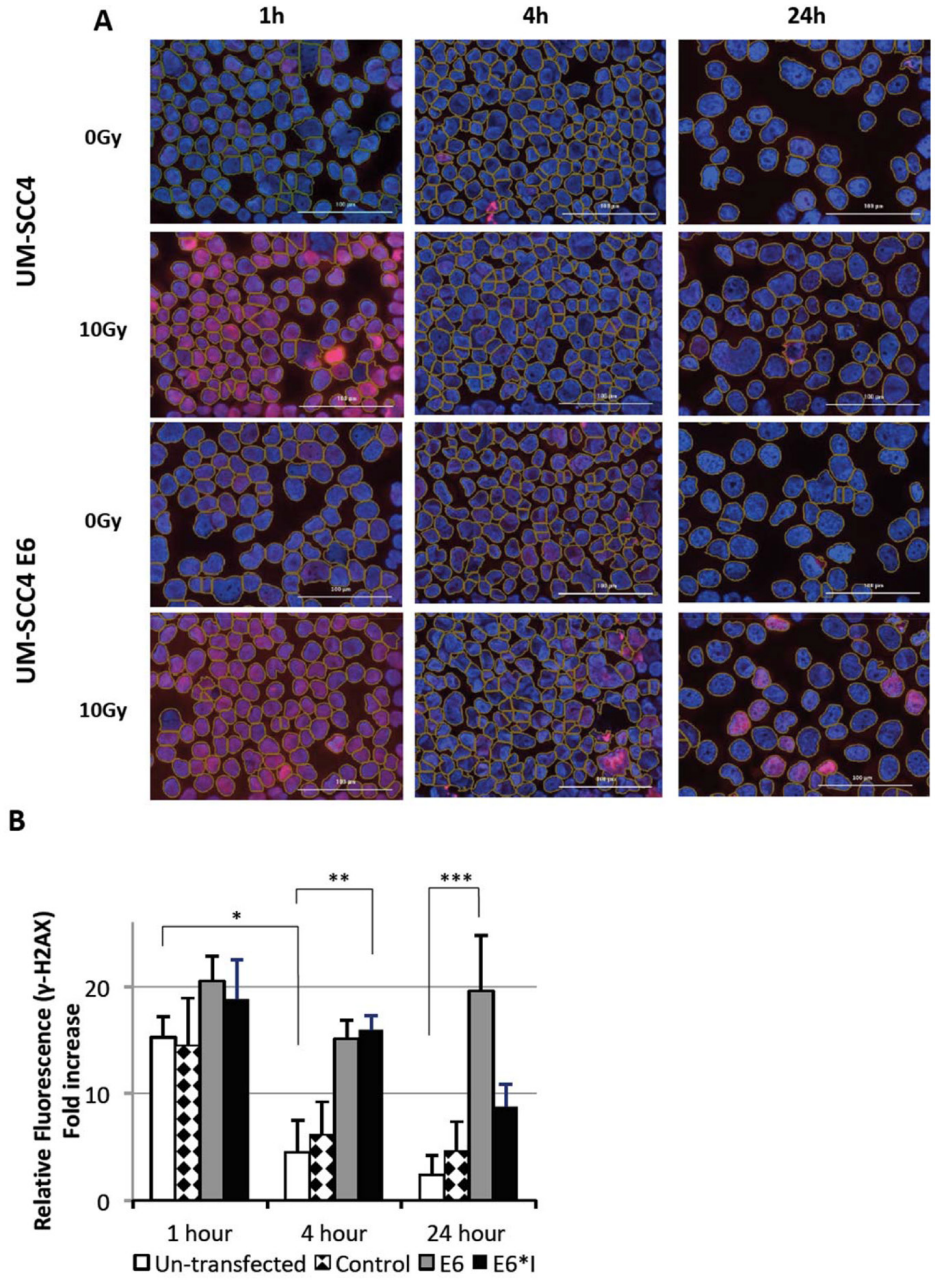
Radiation induced DNA damage measured by γ-H2AX foci formation at specified time point after 10 Gy irradiation. (**A**) Representative images comparing untransfected UM-SCC4 and HPV-E6 transfected UM-SCC4. Magnification: ×20, scale bar = 100 μm. (**B**) Column graph demonstrate the quantitative analyze of γ-H2AX foci by relative fold change of fluorescent intensity. at 1 hour, 4 hours and 24 hours post irradiation. ^*^ *P* = 0.0001, ^**^ *P* = 0.024, ^***^ *P* = 0.021.

There was a significant difference in γ-H2AX foci formation between the untransfected UM-SCC4 cells and E6 transfected UM-SCC4 cells (Figure 6A). From quantitative measurement shown in Figure 6B, the induction of γ-H2AX peaked at 1 hour after 10 Gy radiation at more than 15-fold in all cell lines (untransfected UM-SCC4, plasmid transfected control, E6 total protein transfected and E6*I transfected). At 4 hours, the γ-H2AX expression in both untransfected and plasmid transfected control reduced significantly (*P* < 0.01) and returned to close to the minimum level by 24 hours. The γ-H2AX intensity in HPV 16 E6 transfected cells persisted up to 24 hours post 10 Gy radiation. The difference in the level of γ-H2AX expression between HPV 16 E6 transfected and untransfected cells at 4 hours (*p* = 0.024) and 24 hours (0.021) post radiation was significant.

## DISCUSSION

In this *in vitro* study, the HPV-positive UPCI-SCC-099 cell line is more radiosensitive than the HPV-negative cell line UPCI-SCC-089 as measured by cell proliferation study and clonogenic assay. The results are consistent with a previous study of a panel of head and neck cancer cell lines showing enhanced radiosensitivity using clonogenic assay [12]. In that study, the survival fraction after 3 Gy of radiation was 0.2827 in HPV-positive cell lines and 0.4455 in HPV-negative cell lines. To explore the difference in radiosensitivity, we examined the level of radiation induced DSB by measuring the γ-H2AX signal. There was a significant difference in radiation induced DSB in HPV-positive and HPV-negative cells. For HPV-negative UPCI-SCC-089 cell line, the γ-H2AX signal peaked at 4 hours after radiation then reduced dramatically at 24 hours. This indicated that the cells were able to repair the radiation induced DSB within 24 hours. In contrast, HPV-positive UPCI-SCC-099 showed prolonged γ-H2AX foci formation up to 48 hours after radiation. Therefore these cells were less able to repair the radiation induced DSBs and hence more likely to undergo mitotic cell death during the cell cycle.

We have previously shown that the HPV oncoprotein E6*I enhanced the radiosensitivity of UM-SCC4 with approximately eightfold lower surviving cell fraction after 10 Gy [17]. Flow cytometric analyses showed that irradiated E6^*^I expressing cells had a much higher G2M: G1 ratio than control cells, indicating that, after G2, cells were diverted from the cell cycle to programmed cell death. To explore the role of HPV oncoprotein E6 in DNA repair process, we measured the γ-H2AX foci formation in the UM-SCC4 cells with or without E6 oncoprotein. We have found that 10 Gy of radiation induced a 15-fold increase in γ-H2AX foci formation at 1 hour in the untransfected UM-SCC4, plasmid transfected control, E6 total protein transfected and E6*I transfected cells. The γ-H2AX formation then reduced significantly at 4 hours and dropped to a minimum level by 24 hours in the untransfected and plasmid transfected control cells. In contrast, γ-H2AX formation in HPV E6 transfected UM-SCC4 cells persisted up to 24 hours post radiation. These findings suggest that the E6 oncoprotein reduced the ability to repair radiation induced DSB. These cells continue to progress through the cell cycle despite DNA damage, which normally stops this progression and induces activation of DNA repair mechanisms. The number of γ-H2AX foci is regarded as a sensitive and quantitative surrogate marker for the number of radiation-induced DSBs [18]. Persistence of γ-H2AX level after radiation is a sign of lethal damage [7–10]. γ-H2AX has been increasingly used to investigate the correlation between cellular radiosensitivity and the presence of residual foci [7, 19]. Meneceur et al. demonstrated the validity of residual γ-H2AX foci as a marker of radiosensitivity in head and neck cancers [20]. γ-H2AX expression is also considered to be a candidate biomarker for clinical setting to predict treatment outcomes [9, 20–22].

The classical features of apoptosis are cell shrinking, membrane blubbing and nuclear condensation. These distinct apoptotic features can be observed by real time HoloMonitor. In our study, the HPV-positive UPCI-SCC-099 cells showed an increase in cell thickness and motility after radiation exposure. These suggest that the cells were undergoing apoptotic death and the alteration of the cellular membrane may enhance the cellular movement of UPCI-SCC-099 cells. The enhanced cell motility is due to disruption of the actin-membrane interactions by radiation, initiating the membrane blubbing and generating force to enhance cell motility [23, 24]. In contrast, the HPV-negative UPCI-SCC-089 cells exhibited cell flattening and enlargement, which are the common cytological features of cell cycle blockage [25]. These data from real time tracking suggest that HPV-positive OPSCC had enhanced apoptotic death and increased motility after radiation.

## MATERIALS AND METHODS

### Cell cultures

Human OPSCC cell lines UM-SCC4 (HPV-negative), UPCI-SCC-089 (HPV-negative), and UPCI-SCC-099 (HPV-positive) were used in this study [26, 27]. In addition, two HPV-negative UM-SCC4 cell lines transfected with the HPV E6 oncoprotein (UM-SCC4 E6 total and UM-SCC4 E6*I) previously established by the authors’ group were also used in this study [18]. All cell cultures were maintained in exponential growth in Advanced RPMI 1640 medium (GIBCO, ThermoFisher Scientific, Inc.) with 5% fetal bovine serum (FBS). The cells were cultured at 37°C in a humidified atmosphere containing 5% CO_2_. The cell lines were routinely screened for mycoplasma.

### Radiation exposure

The cells were irradiated in suspension using a blood product irradiator (Gammacell 3000 Elan, Nordion, Ontario, Canada) at single doses of 0, 2, 5, and 10 Gy. The irradiator had a 137Cs source and a cylindrical canister. The dose rate was 3.2 Gy per minute at the center of the canister. Radiation dose homogeneity was estimated at 10% using existing clinical depth-dose data for this irradiator.

### Clonogenic cell survival assay

Following radiation, the UPCI-SCC-089 and UPCI-SCC-099 cells in suspension were counted, diluted, and seeded in T25 flasks for clonogenic assay. The density of the cells (500–250,000 cells per flask) was adjusted to allow for plating efficiency and clonogenic survival. The cells were plated in triplicate and incubated in Advanced RPMI 1640 with 10% FBS at 37°C for 17–21 days. The colonies were fixed and stained for 30 minutes with 0.3% methylene blue in methanol. Colonies containing more than 50 cells were counted, and the survival fractions were used to generate radiation survival curves.

### γ-H2AX foci detection

Immunofluorescent assay was used for γ-H2AX detection. Irradiated cells were spun onto Superfrost glass slides at 1 hour, 4 hours, 24 hours, and 48 hours after radiation. The cells were fixed in formalin acetic acid for 2 hours at room temperature and permeabilized in 1% Triton X-100, followed by washing in Tris-Buffered Saline + Tween (TBST) and blocked with DAKO blocking solution (DAKO ×0909). The cells were incubated with anti-γ-H2AX antibody (Phospho-Histone H2A.× Rabbit mAb #9718, Cell Signaling) for 2 hours at room temperature. Concentrations of anti-γ-H2AX were 1:500 for UM-SCC4 and 1:300 for UPCI-SCC-089 and UPCI-SCC-099. The cells were incubated with 1:500 dilution secondary antibody (Anti-rabbit IgG Alexa Fluor 594 Conjugate #8889, Cell Signaling) for 1 hour at room temperature. The slides were then washed and the nucleus was counterstained with DAPI for 15 minutes at room temperature. A Cytation3 image reader (BioTek Instrument, Inc.) was used to capture the fluorescent images using an LED tube at 365 nm for nuclei staining of DAPI and a 590-nm LED tube for γ-H2AX. The γ-H2AX foci were quantitated using Gen5 Microplate Reader and Image Software (BioTek Instrument, Inc.). At least five view images were captured randomly from each sample; 200 to 800 cells were captured in each image depending on the cell line, time points, and treatment. Each nucleus was marked and the mean and peak of intensity of γ-H2AX and DAPI were calculated with Gen5. The final γ-H2AX expression was determined as the average fluorescent intensity from all cells in each sample. The change of γ-H2AX expression post radiation exposure was compared with non-irradiated cells. The threshold of background fluorescent was set at 500.

### Morphological changes after radiation by digital holographic microscopy

After irradiation, 2–4 × 10^5^ cells/well were seeded in 6-well plate for 4 hours to allow the cells to attach. Time-lapse holographic cell images from five representative frames in each well were captured and recorded every 30 minutes for 48 hours using HoloMonitor™ M4 (Phase Holographic Imaging AB, Lund, Sweden). Cell number counts, cell sizes, cell volume (μm^3^), cell thickness (μm) and cell movements were analyzed with H-studio software 2.7.3 (Phase Holographic Imaging AB, Lund, Sweden).

### Statistical analysis

Unless otherwise stated, data in the charts represent the mean and standard error of the experimental groups from at least three independent experiments. The statistical analysis was performed using Student’s *t*-tests. The data are presented as mean ± standard error of mean (SEM). A probability level of a *P* value of < 0.05 was considered significant.

## CONCLUSIONS

In conclusion, this study supports a role for E6 in the enhancing the radiosensitivity of HPV-positive OPSCC cells. We have demonstrated that the HPV oncoprotein E6 played a role in enhancing the radiosensitivity by affecting the repair of radiation induced DSB and enhanced apoptosis formation.

## SUPPLEMENTARY MATERIALS







## Abbreviations

HPV: Human papillomavirus
OPSCC: Oropharyngeal squamous cell carcinoma
DSB: double-strand break
SF2: Survival after 2 Gy
FBS: Fetal bovine serum
