# Supplementary material for: The human papillomavirus confers radiosensitivity in oropharyngeal cancer cells by enhancing DNA double strand break
Source: Oncotarget. 2020 Apr 21;11(16):1417–26. doi: 10.18632/oncotarget.27535 (PMC7185066; doi:10.18632/oncotarget.27535)
Supplement: Supplementary file 1 [file oncotarget-11-1417-s001.pdf]

The human papillomavirus confers radiosensitivity in oropharyngeal cancer cells by enhancing DNA double strand break

SUPPLEMENTARY MATERIALS

Supplementary Video 1: See Supplementary Video 1

Supplementary Video 2: See Supplementary Video 2
